# Supplementary material for: Inferring the heritability of large-scale functional networks with a multivariate ACE modeling approach
Source: Netw Neurosci. 2021 Jun 3;5(2):527–48. doi: 10.1162/netn_a_00189 (PMC8233119; doi:10.1162/netn_a_00189)
Supplement: Supplementary file 1 [file netn-05-527-s001.pdf]

Ribeiro, F. L., dos Santos, F. R. C., Sato, J. R., Pinaya, W. H. L. & Biazoli, C. E. (2021). Supporting information for "Inferring the heritability of large-scale functional networks with a multivariate ACE modeling approach." *Network Neuroscience.* Advance publication. https://doi.org/10.1162/netn_a_00189

Supplementary material

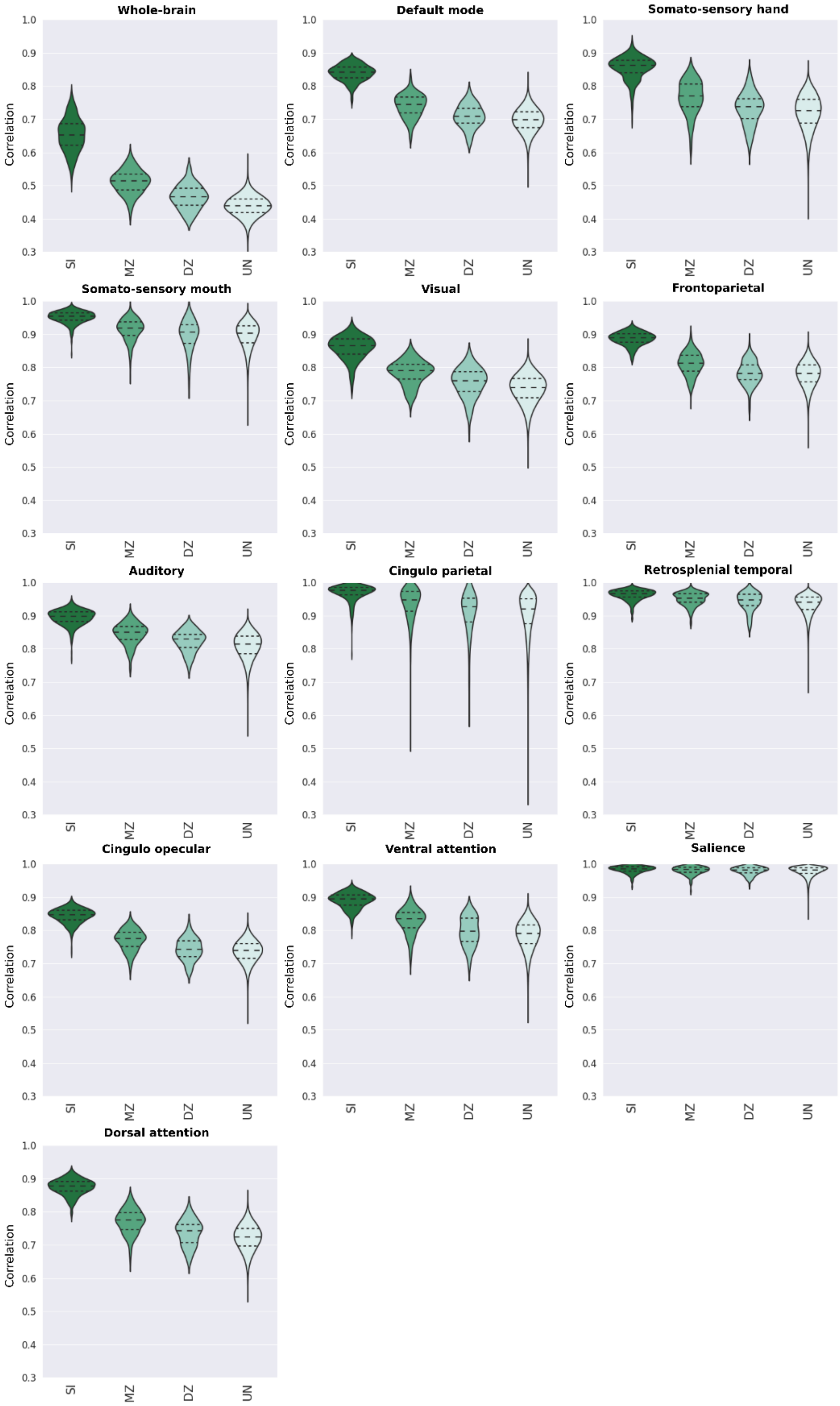

Supplementary Figure 1 - Distribution of correlation coefficients between pairs of functional connectivity matrices for the whole-brain and individual functional networks defined for "Gordon" parcellation schema. Pearson's correlation scores were determined from pairs of connectivity matrices (REST1 × REST2), and they were grouped based on individuals' genetic relationship. Hence, violin plots show the distribution of the correlation scores between pairs of matrices of the same individual (SI), monozygotic twin (MZ), dizygotic twin (DZ) and unrelated individuals (UN).

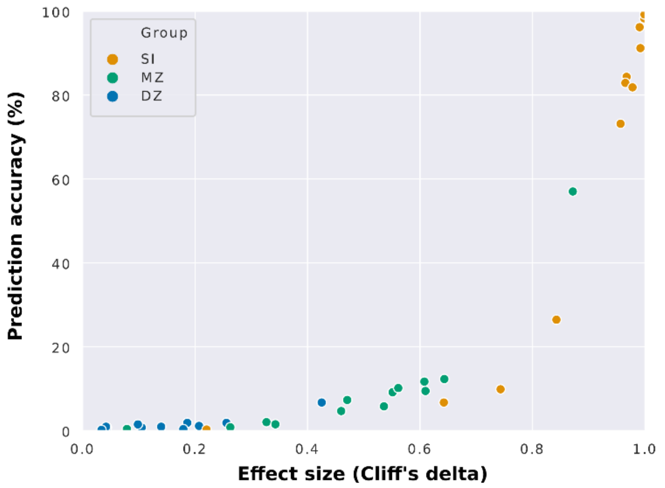

Supplementary Figure 2 - Dependence of connectome-based predictions on effect size using "Gordon" parcellation. Here, the mean prediction accuracies from all functional networks (whole-brain included) were plotted as a function of the effect size of the difference between the group of interest (same individual – SI, monozygotic twins – MZ, or dizygotic twins – DZ) and unrelated individuals.

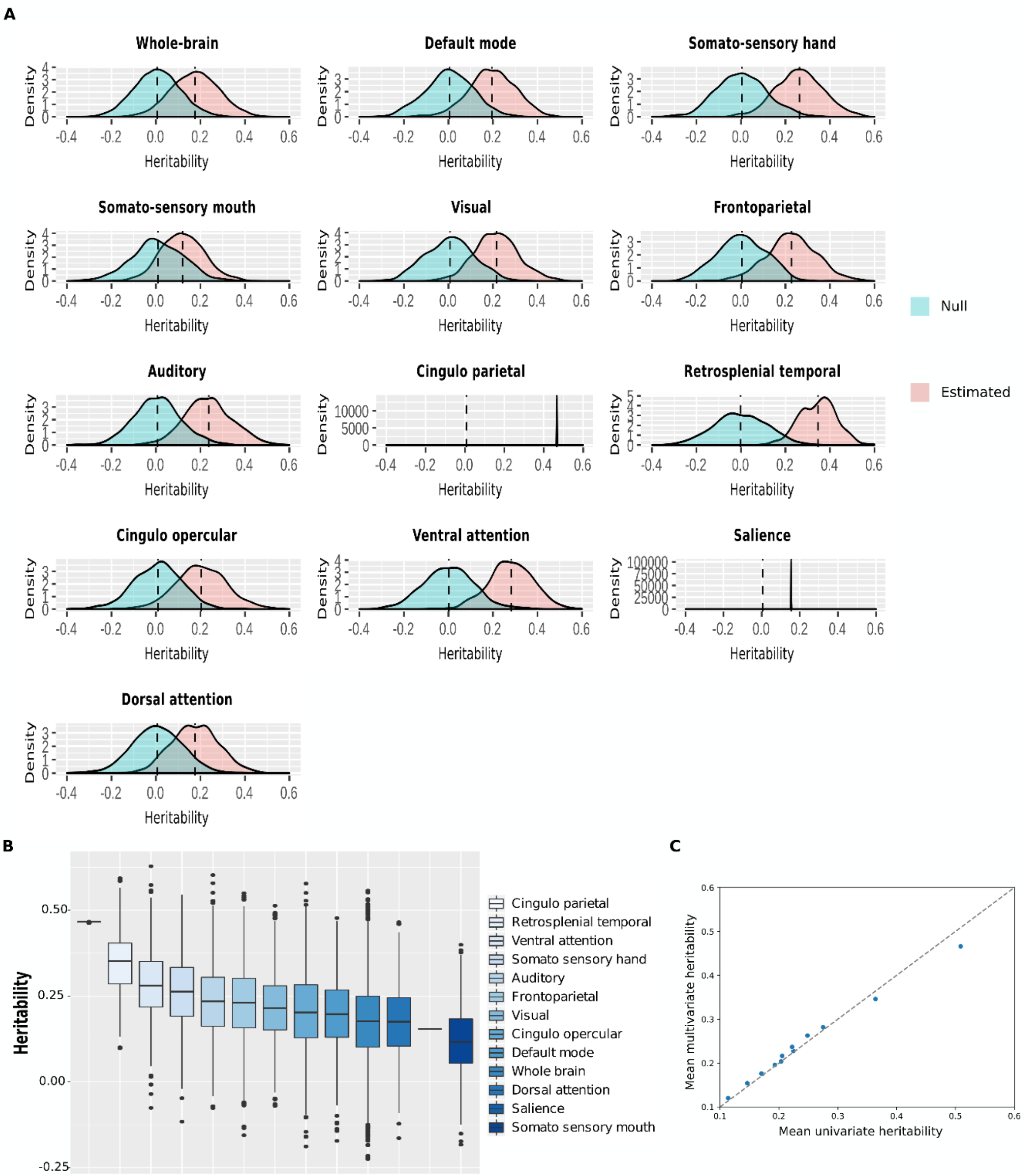

Supplementary Figure 3 - Heritability distributions for each functional network using ‘Gordon’ parcellation. A) Heritability estimates and null distributions for each functional network. **B)** Heritability estimates distributions are shown from the most heritable (cingulo parietal) to the least heritable (somato sensory mouth). **C)** Comparison of the mean heritability found with multivariate ACE models versus univariate ACE models for all functional networks.

Supplementary Table 1 - Network-based individual and twin identification accuracies using "Gordon" and "Shen" parcellation schemas. Note: Functional networks were *a priori* defined. For "Gordon" parcellation schema, 47 ROIs were not assigned to any specific functional network. Thus, these ROIs were only considered for whole-brain functional connectivity based identifications. Mean identification accuracies for individual and twin identification analyses for all functional networks (whole-brain included). Mean identification for individual prediction was determined from two combinations of 'database' and 'target' sets (RESTX × RESTY, where X and Y ∈ {1, 2} and X≠Y), while the mean twin identification was determined from four combinations (RESTX × RESTY, where X and Y ∈ {1, 2}).

	Prediction accuracy ± SD (%)					
Network, number of ROIs	Individual		Monozygotic twin		Dizygotic twin	
	Mean	Std	Mean	Std	Mean	Std
"Gordon"						
Whole-brain, 333	99.87	0.13	57.01	0.83	6.72	1.40
Default mode, 41	98.29	0.13	11.69	2.50	1.87	1.24
Smhand, 38	84.34	0.39	7.32	1.11	0.75	0.00
Smmouth, 8	26.45	0.39	0.81	0.29	0.37	0.37
Visual, 39	82.89	0.53	9.45	0.88	1.87	0.65
Frontoparietal, 24	91.18	1.45	4.67	0.61	0.75	0.53
Auditory, 24	73.16	0.53	5.79	0.34	0.00	0.00
Cingulo Parietal, 5	9.87	0.66	2.03	1.22	0.93	0.62
Retrosplenial Temporal, 8	6.71	0.66	1.52	0.67	0.37	0.65
Cingulo Opercular, 40	96.18	0.39	9.15	1.09	1.49	1.06
Ventral Attention, 23	81.84	0.53	10.16	0.81	0.93	0.81
Salience, 4	0.26	0.00	0.41	0.70	0.19	0.32
Dorsal Attention, 32	99.21	0.00	12.30	2.31	1.12	0.65
"Shen"						
Whole-brain, 268	97.76	0.39	57.22	2.61	8.96	2.36
Medial frontal, 29	93.95	0.53	21.14	2.23	3.54	0.97
Frontoparietal,34	91.84	0.26	17.17	2.36	2.05	1.33
Default mode, 20	61.58	0.00	5.59	1.20	1.31	0.62
Subcortical-cerebellum, 90	87.24	0.13	28.56	1.50	2.05	1.53
Motor, 50	79.08	0.92	18.09	0.93	2.24	0.53
Visual I, 18	63.95	0.26	7.72	0.86	2.61	0.83
Visual II, 9	15.13	0.66	2.24	0.93	1.68	1.62
Visual association, 18	55.53	0.00	5.69	1.97	0.93	0.62

Supplementary Table 2 - Correlation score between the number of nodes and the average identification scores.

	Pearson correlation	Significance (p-value)
"Gordon"		
SI	0.40	0.17
MZ	0.99	5.87E-10
DZ	0.97	7.66E-8
"Shen"		
SI	0.52	0.15
MZ	0.95	6.27E-5
DZ	0.92	3.91E-4

Supplementary Table 3 - Effect size of the difference among groups of correlation scores using Cliff's delta.

	Cliff's delta		
	SI vs UN	MZ vs UN	DZ vs UN
"Gordon"			
Whole-brain	1.00	0.87	0.43
Default mode	1.00	0.61	0.19
Smhand	0.97	0.47	0.11
Smmouth	0.84	0.26	0.04
Visual	0.97	0.61	0.26
Frontoparietal	0.99	0.46	0.04
Auditory	0.96	0.54	0.21
Cingulo Parietal	0.74	0.33	0.04
Retrosplenial Temporal	0.64	0.34	0.18
Cingulo Opercular	0.99	0.55	0.10
Ventral Attention	0.98	0.56	0.14
Salience	0.22	0.08	0.03
Dorsal Attention	1.00	0.64	0.21
"Shen"			
Whole-brain	1.00	0.89	0.45
Medial frontal	0.99	0.73	0.34
Frontoparietal	0.99	0.69	0.26
Default mode	0.95	0.55	0.24
Subcortical-cerebellum	0.98	0.81	0.31
Motor	0.95	0.68	0.24
Visual I	0.94	0.61	0.36
Visual II	0.73	0.38	0.19
Visual association	0.89	0.52	0.28

Supplementary Table 4 – Mean heritability (multivariate ACE model) and confidence interval of each functional network.

Network	Mean	Lower bound (2.5 percentile)	Upper bound (97.5 percentile)
"Gordon"			
Whole-brain	0.18	-0.04	0.39
Default mode	0.20	-0.02	0.39
Smhand	0.26	0.06	0.47
Smmouth	0.12	-0.06	0.31
Visual	0.22	0.03	0.42
Frontoparietal	0.23	0.00	0.45
Auditory	0.24	0.03	0.45
Cingulo Parietal	0.47	0.47	0.47
Retrosplenial Temporal	0.35	0.19	0.50
Cingulo Opercular	0.20	-0.02	0.44
Ventral Attention	0.28	0.08	0.47
Salience	0.15	0.15	0.15
Dorsal Attention	0.18	-0.01	0.38
"Shen"			
Whole-brain	0.24	0.03	0.45
Medial frontal	0.31	0.12	0.49
Frontoparietal	0.27	0.08	0.47
Default mode	0.22	0.02	0.43
Subcortical-cerebellum	0.20	-0.01	0.40
Motor	0.30	0.08	0.52
Visual I	0.25	0.07	0.45
Visual II	0.37	0.24	0.51
Visual association	0.25	0.07	0.42

Supplementary Table 5 - Independent t-test p-values of comparisons among functional networks as defined "Shen" parcellation. Note: Alpha-level corrected for multiple comparisons is equal to 0.0015625.

	Whole-brain	Medial frontal	Frontoparietal	Default mode	Subcortical-cerebellum	Motor	Visual I	Visual II	Visual association
Whole-brain		7.29E-81	4.46E-23	4.51E-09	1.57E-28	5.54E-60	0.000613	6.3E-282	0.006782
Medial frontal			1.86E-13	1.61E-80	3E-111	0.055264	1.56E-37	3.92E-55	1.84E-42
Frontoparietal				2.75E-33	1.35E-55	6.16E-07	1.58E-07	1.1E-111	2.77E-09
Default mode					6.11E-05	2.75E-58	1.72E-13	1.1E-236	3.92E-12
Subcortical-cerebellum						4.13E-84	7.06E-30	3E-278	3.08E-28
Motor							1.28E-23	6.83E-59	6.06E-27
Visual I								1.6E-174	0.535596
Visual II									1.2E-189
Visual association									

Supplementary Table 6 - Independent t-test p-values of comparisons among functional networks as defined "Gordon" parcellation. Note: Alpha-level corrected for multiple comparisons is equal to 0.05/78=0.000641026.

	Whole-brain	Default mode	Somato-sensory hand	Somato-sensory mouth	Visual	Frontoparietal	Auditory	Cingulo Parietal	Retrosplenial Temporal	Cingulo Opercular	Ventral Attention	Salience	Dorsal Attention
Whole-brain		1.14E-08	2.2E-130	1.37E-55	1.16E-30	1.16E-47	4.75E-66	0	0	4.21E-15	1.9E-192	0.527916	0.919909
Default mode			2.02E-43	3.03E-60	6.94E-06	1.01E-10	9.91E-18	0	9.3E-217	0.112817	1.02E-72	0.206359	2.22E-05
Somato-sensory hand				1.8E-177	2.06E-23	9.84E-13	7.23E-08	0	2.66E-78	1.03E-31	3.81E-05	0.001319	9.58E-70
Somato-sensory mouth					8.94E-98	5E-104	2.2E-126	0	0	2.35E-65	3.4E-229	0.260407	1.42E-34
Visual						0.0189	8.93E-06	0	1.2E-181	0.008015	7.98E-47	0.046014	1.42E-18
Frontoparietal							0.056683	0	3.4E-136	3.02E-06	3.16E-29	0.039849	1.58E-25
Auditory								0	4.8E-125	2.95E-11	9.33E-22	0.014828	2.16E-36
Cingulo Parietal									0	0	0	0	0
Retrosplenial Temporal										3.9E-181	1.11E-52	2.84E-13	3.5E-262
Cingulo Opercular											7.41E-56	0.171995	1.98E-08
Ventral Attention												5.61E-05	3E-105
Salience													0.506939
Dorsal Attention													

Supplementary Table 7 – Mean heritability (univariate ACE model) and confidence interval of each functional network

Network	Mean	Lower bound (2.5 percentile)	Upper bound (97.5 percentile)
"Gordon"			
Whole-brain	0.17	-0.46	0.81
Default mode	0.19	-0.46	0.83
Smhand	0.25	-0.32	0.86
Smmouth	0.11	-0.65	0.87
Visual	0.21	-0.38	0.81
Frontoparietal	0.22	-0.40	0.94
Auditory	0.22	-0.39	0.87
Cingulo Parietal	0.51	0.09	0.92
Retrosplenial Temporal	0.36	-0.27	0.90
Cingulo Opercular	0.20	-0.47	0.88
Ventral Attention	0.28	-0.35	0.86
Salience	0.15	-0.15	0.61
Dorsal Attention	0.17	-0.48	0.81
"Shen"			
Whole-brain	0.23	-0.40	0.86
Medial frontal	0.31	-0.27	0.82
Frontoparietal	0.27	-0.33	0.88
Default mode	0.22	-0.40	0.85
Subcortical-cerebellum	0.19	-0.43	0.85
Motor	0.29	-0.33	0.92
Visual I	0.24	-0.32	0.76
Visual II	0.36	-0.16	0.79
Visual association	0.25	-0.28	0.86
